# Genome Sequence of Oxalobacter formigenes Strain SSYG-15

**DOI:** 10.1128/MRA.01059-19

**Published:** 2019-10-17

**Authors:** Ning-yun Sun, Yuan Gao, Hong-jing Yu

**Affiliations:** a Pharmaceutical R&D Center, SPH Sine Pharmaceutical Laboratories Co., Ltd., Shanghai, China; b Shanghai Engineering Research Center of Innovative Probiotic Drugs, Shanghai, China; Georgia Institute of Technology

## Abstract

Colonization of the intestine with Oxalobacter formigenes reduces urinary oxalate excretion and lowers the risk of forming calcium oxalate kidney stones. Here, we report the genome sequence of Oxalobacter formigenes SSYG-15, a strain isolated from a stool sample from a healthy Chinese boy.

## ANNOUNCEMENT

Oxalobacter formigenes, an anaerobe that is extremely sensitive to oxygen with substrate specificity for oxalate, was first isolated in 1985 by Milton J. Allison and then was identified as a new genus and species (1). This microorganism has a potential effect on oxalate metabolism in the human body (2, 3).

We isolated one Oxalobacter formigenes strain from the feces of a healthy Chinese boy who resided in Shanghai, China. We serially diluted 1 g of the feces sample and then spread it over ATCC medium 1352 with 7% agar and incubated it in an anaerobic environment at 37°C for 7 days. One white colony growing on the plates after 7 days of incubation was identified as Oxalobacter formigenes (99.6% identity to the 16S rRNA gene of Oxalobacter formigenes OXCC13) based on 16S rRNA gene sequencing. This strain was named Oxalobacter formigenes SSYG-15 (4), and it grew optimally at 37°C, with a doubling time of 7 days.

For genomic DNA extraction, a single colony was inoculated in ATCC medium 1352 broth and cultured in an anaerobic environment at 37°C until saturation. Genomic DNA was isolated using the MagAttract high-molecular-weight (HMW) DNA kit (Qiagen) according to the manufacturer’s instructions (for Gram-negative bacteria). Library preparation was performed with the SMRTbell template prep kit 1.0 (Pacific Biosciences, USA), followed by single-molecule real-time (SMRT) sequencing on the Pacific Biosciences RS II platform (5).

A single SMRT cell produced a total of 2.52 Gb in 56,490 polymerase reads, and the *N*_50_ value of 66.16 kb was used for *de novo* assembly with the PacBio SMRT Analysis 5.1.0 assembly protocol, resulting in one circular chromosome with a single contig of 2,491,742 bp. The chromosomal contig showed an average G+C content of 49.56%.

Genome annotation was completed by submission to the NCBI Prokaryotic Genome Automatic Annotation Pipeline (PGAAP). A total of 2,255 predicted genes, 6 rRNAs, 47 tRNAs, and 4 noncoding RNAs were obtained. AntiSMASH2.0.2rc1 analysis (6) resulted in the prediction of a secondary metabolite biosynthesis prediction gene cluster of formic acid. Meanwhile, antibiotic resistance gene analysis with Resistance Gene Identifier (RGI) (7) predicted a gene associated with resistance to macrolide, penicillin, trimethoprim, aminoglycoside, and chloramphenicol. Default parameters were used for all software programs unless otherwise stated.

Oxalobacter formigenes strain SSYG-15 was archived in the China Center for Type Culture collection in 2016 and was assigned CCTCC number M2016183.

### Data availability.

The complete genome sequence of Oxalobacter formigenes SSYG-15 is available from GenBank under the accession number CP042242. The raw sequence reads have been deposited in the NCBI Sequence Read Archive under the BioProject number PRJNA556554 and accession number SRR10007720.
